# Quality of Life and Body Mass Index Changes Three Years After Laparoscopic Sleeve Gastrectomy in Taif City, Saudi Arabia

**DOI:** 10.7759/cureus.32754

**Published:** 2022-12-20

**Authors:** Amjad M Jawhari, Abdulaziz M. Alrashed, Hussam Alghamdi, Abdulaziz S. AlOtaibi, Khaled Alshareef, Khalid M Alzahrani

**Affiliations:** 1 College of Medicine, Taif University, Taif, SAU; 2 Department of Surgery, King Abdulaziz Specialist Hospital, TAIF, SAU; 3 College of Medicine, Department of Surgery, Taif University, Taif, SAU

**Keywords:** bariatric & metabolic surgery fbms, gerd, obesity, bariatric surgery, sleeve gastrectomy

## Abstract

Background

Obesity has become a major global health challenge, and its prevalence has tripled in the last four decades. Impaired quality of life (QoL) is a strong incentive for severely obese patients to seek help. Sleeve gastrectomy (SG) is the most frequently practiced bariatric procedure worldwide. This study aimed to investigate the QoL and changes in body weight three years post laparoscopic SG.

Methods

A cross-sectional, observational study was performed in outpatient clinics in Taif city, Saudi Arabia. The study included 147 adult patients who underwent SG at least three years before inclusion in the study. Data were collected using a questionnaire designed based on the validated Bariatric quality of life (BQL) and Gastroesophageal Reflux Disease Health-Related Quality of Life (GERD-HRQoL) surveys.

Results

All patients were suffering from class I, class II, or class III obesity before undergoing SG. Three years post-surgery, 72.8% reached their normal weight or were overweight (P<0.001). The mean± SD BMI (45± 7.0 kg/m^2^) significantly decreased to 26.8± 4.6 kg/m^2^ (P<0.001). Most of the participants (78.2%) achieved an excess weight loss percent (EWL%) of 75% or more. The mean± SD BQL score was 45.5± 5.2 points and the median (IQR) GERD-HRQoL score was 7 (15). Higher EWL% was significantly associated with a higher BQL score (P=0.041).

Conclusions

The current study revealed a better quality of life among patients experiencing higher rates of excess weight loss percent (EWL%) after three years of sleeve gastrectomy as compared to other patients.

## Introduction

Obesity continues to be a serious health problem with increasing prevalence worldwide. Between 1975 and 2016, the global prevalence of obesity nearly tripled. In 2016, the World Health Organization (WHO) reported that more than 650 million adults suffer from obesity. This number represents around 13% of the global adult population [1].

Obesity leaves individuals suspectable to multiple chronic diseases, including type two diabetes mellitus (T2DM), insulin resistance, cardiovascular diseases, hypertension, stroke, hypercholesterolemia, musculoskeletal disorders, osteoarthritis, chronic kidney disease, lower urinary tract symptoms, including urinary incontinence, pulmonary diseases, sleep apnea, fatty liver, gall bladder disease, polycystic ovary syndrome, irregular menstruation, cancers, and infertility [2-4].

Poor quality of life (QoL) is the main motivator that pushes obese patients to seek medical attention for their condition. Therefore, it is safe to consider the changes in QoL as the main measurement of obesity treatment efficacy [5].

Weight reduction, improvement of QoL, and alleviating comorbidities related to obesity are considered the main goals of bariatric surgery that have been proven to be the gold-standard treatment for morbid obesity. Literature reported that bariatric surgery is a therapeutic option that results in long-lasting and effective weight loss [6].

Laparoscopic sleeve gastrectomy is the most popular bariatric surgery for obesity management in the United States and worldwide [7]. The main benefits of bariatric surgery include promoting substantial weight loss and resolution of T2DM and other obesity-related comorbidities. Weight loss, in general, will lead to short-term improvement in the individual's overall health and will help prevent future metabolic disorders and cardiovascular events [8].

In 1988, sleeve gastrectomy (SG) was first introduced as part of the biliopancreatic diversion duodenal switch procedure. Later on, it was performed laparoscopically and soon became a standalone procedure. The main reason for laparoscopic SG's growing popularity is the simplicity of its technique compared to other surgeries such as the laparoscopic Roux-en-Y gastric bypass (LRYGB). After SG, the reduced stomach size results in increased satiety and decreased appetite and induces weight loss [9].

In the first international consensus summit for SG, all experts agreed that SG is indicated for patients with a body mass index (BMI) > 60 kg/m^2^. The majority of experts agreed that SG is the primary procedure for patients with BMI > 40 or > 35 and comorbidities such as T2DM, cirrhosis, and sleep apnea. However, the experts agreed that the incidence of gastroesophageal reflux disease (GERD) after SG is significantly more frequent [10].

Short-term (one year) and medium-term (two to three years) improvements in a variety of patient-reported outcomes are associated with SG, although long-term (5 years) findings on QoL after SG are scarce [11-13].

The bariatric quality of life (BQL) questionnaire was developed in 2005 by Weiner et al. to evaluate the quality of life of patients undergoing bariatric surgery [14]. The questionnaire was validated in a study published in 2009 [15]. The BQL gives results that are very similar to the results of the widely used short-form 12-item health survey (SF12) [16].

The symptoms and complications of GERD affect the general health, social life, and emotions of patients as well as the overall health-related quality of life (HRQoL), as it causes frequent sleep interruptions, generalized body pain, inconveniences during work, impaired sex life, in addition to difficulties during physical and social activities [17].

Currently, obesity is a major public health problem in Saudi Arabia, and several challenges are being faced to control its incidence and prevalence. Around 70% of Saudi people experience obesity-related complications such as T2DM, hypertension, hyperlipidemia, and sleep apnea [18].

In order to minimize the risk of obesity-associated comorbidities and improve patients’ QoL, the Saudi clinical practice guidelines and the American Society for Metabolic and Bariatric Surgery recommend bariatric surgery for obese patients with BMI ≥ 40 kg/m^2^ or ≥35 kg/m^2^ with comorbidities and there has been a noticeable increase in the number of obese patients who undergo bariatric surgery in Saudi Arabia. It is estimated that more than 15,000 bariatric surgeries are performed yearly in Saudi Arabia mainly due to their effectiveness in weight loss, especially over a period of 12 to 18 months post-surgery [19].

The current study was conducted to investigate the change in body weight, as well as the change in patients’ QoL, at three years post laparoscopic sleeve gastrectomy.

## Materials and methods

Study design and setting

This was a cross-sectional, observational, survey-based study conducted in the outpatient surgical clinics at King Abdulaziz Specialist Hospital (KAASH), Taif City, Saudi Arabia. The study population included 147 adult bariatric patients attending outpatient surgical clinics at KAASH. The inclusion criteria were: adult patients aged between 18 and 65 years and patients who underwent a laparoscopic gastric sleeve operation at least three years ago. The exclusion criteria were: patients who had gastric bypass and patients who were unwilling to take part in the study. Data were collected using a self-administered questionnaire. Patients were allowed enough time to fill out the questionnaire privately and confidentially. Collected data were entered into a Microsoft Excel sheet (Microsoft Corporation, Redmond, WA) for validation. A self-administered designed Arabic questionnaire was used for data collection. The questionnaire included three main sections: a section to collect socio-demographic characteristics of the participants, one section representing the Bariatric Quality of Life (BQL) questionnaire, and another section representing the Gastroesophageal Reflux Disease Health-Related Quality of Life (GERD-HRQoL) survey.

The BQL is a 30-item validated questionnaire used to evaluate patients’ quality of life (QoL) after bariatric surgery. The total BQL index score is calculated by summing up all scores of all questions, and the score ranges from 0 to 78 points where higher scores represent better QoL [10].

The GERD-HRQoL questionnaire includes 10 items. Each item can be given a score from 0 to 5 giving a maximum total score of 50 where higher scores indicate worse symptoms [20]. This questionnaire was used because GERD is a common health problem among bariatric surgery patients that may significantly affect their QoL.

Total weight loss (TWL%) was calculated using the formula TWL%= (W1-W2)/W1*100, where W1 is the body weight before the surgery and W2 is the body weight after the surgery.

Excess weight loss (EWL%) was calculated using the formula EWL%= (W1-W2)/(W1-(Ideal body weight)*100, where W1 is the body weight before the surgery and W2 is the body weight after the surgery. All patients who underwent SG prior to March 2019, with a follow-up duration of three years at least, were included in the study. Validated data were analyzed using the Statistical Package for Social Sciences (SPSS), version 28 (IBM Corp., Armonk, NY). Descriptive statistical analysis was conducted to present the data where categorical variables were presented as count and percent. Numerical variables were presented as mean and standard deviation± SD if normally distributed while the BQL index score and the GERD-HRQoL score were presented as the median and interquartile range (IQR). The BQL index score was compared between the different subgroups using unpaired t-test or one-way ANOVA as appropriate. GERD-HRQoL scores were compared between the different subgroups using the Kruskal-Wallis test. Categorical variables (e.g. BMI categories) were compared using the χ2 test. A two-sided P-value of < 0.05 was considered statistically significant. Ethical approval was obtained from the Research and Studies Department at the Directorate of Health Affairs, Taif city. The approval number by the institutional review board (IRB) in King Abdulaziz City for Science and Technology (KACST) is 612. All patients participated in the study voluntarily, and they provided written informed consent. All collected data were kept confidential and were used only for research purposes.

## Results

Participants’ characteristics

A total of 147 patients who underwent SG at least three years before participation in the study filled out the questionnaire. Almost one-half of the study population (52.4%) were men. The mean± SD age of participants was 38.8±9.0 years. Before undergoing SG, the majority of the participants (75.5%) had class III obesity, 18.4% had class II obesity, and 6.1% had class I obesity. While after a mean±SD duration of 3.8±0.39 years post-surgery, only 1.4% of the participants had class III obesity, 1.4% had class II obesity, 23.1% had class I obesity, 33.3% were overweight, and 39.5% reached their normal weight. Additionally, 78.2% of the participants reported an excess weight loss percentage (EWL%) of 75% or more. More details are illustrated in Table 1.

**Table 1:** Characteristics of study participants (n=147)

Age (Years)		
Age Mean± SD	38.8	9.03
Variable	Count	Percent (%)
Gender		
Men	77	52.4
Women	70	47.6
BMI Grade Pre-Sleeve Gastrectomy		
Class I obesity (BMI 30-34.9 kg/m^2^)	9	6.1
Class II obesity (BMI 35-39.9 kg/m^2^)	27	18.4
Class III obesity (BMI ≥ 40 kg/m^2^)	111	75.5
BMI Grade Post-Sleeve Gastrectomy		
Underweight (BMI <18.5 kg/m^2^)	2	1.4
Normal Weight (BMI 18.5–24.9 kg/m^2^)	58	39.5
Overweight (BMI 25–29.9 kg/m^2^)	49	33.3
Class I obesity (BMI 30-34.9 kg/m^2^)	34	23.1
Class II obesity (BMI 35-39.9 kg/m^2^)	2	1.4
Class III obesity (BMI ≥ 40 kg/m^2^)	2	1.4
Percentage of Excess Weight Loss (EWL%)		
Less than 50%	4	2.7
50-74.9%	28	19.0
75% or more	115	78.2
Percentage of Total Weight Loss (TWL%)		
Less than 50%	117	79.6%
50-74.9%	30	20.4%
75% or more	-	-

BQL survey results

The mean± SD BQL score was found to be 45.5± 5.2 points out of 78 points. The first part of the BQL survey collected information about health inconveniences experienced by the patients three years after SG. The most commonly reported inconveniences by the study participants included hair loss (68.7%), flatulence (42.9%), vomiting (39.5%), heartburn (39.5%), and nausea (34.0%) while the least reported inconveniences were asthma/sleep apnea (6.8%) and gout (4.8%). The second part of the BQL survey collected information about patients’ quality of life after the surgery.

GERD-HRQoL survey results

The severity of GERD symptoms was assessed using the GERD-HRQoL questionnaire by asking the patients about the severity of each of the typical GERD symptoms as they feel it. Out of 50 points, the median GERD-HRQoL score of the study participants was 7 out of 50 points with an IQR of 15 points where a lower score indicates better health status and lower severity of symptoms.

Factors affecting BQL index score and GERD-HRQoL score

Our analysis has shown no statistically significant correlation between age, preoperative BMI, current BMI, percentage of EWL, and duration since operation with BQL index score or GERD-HRQoL score (P>0.05).

Study participants were categorized according to their gender, EWL% grade, pre-operative BMI grade, current BMI grade, and their BQL and GERD-HRQoL scores were compared accordingly. As per data analysis, no statistically significant differences between BQL and GERD-HRQoL scores in the different subgroups were revealed (P>0.05) except for EWL% grade, which was found to significantly affect the BQL score (P=0.041). More details about the scores in the different subgroups are provided in Table 2.

**Table 2:** BQL score and GERD-HRQoL in the different subgroups * The unpaired t-test was used to compare the score between the study participants according to their gender and preoperative BMI. A one-way ANOVA test was used to compare the scores of the study participants according to their postoperative BMI and EWL% grade. ** The Kruskal-Wallis test was used to compare GERD-HRQoL scores between the study participants' subgroups. ANOVA: analysis of variance; EWL%: excess weight loss percent; GERD-HRQoL: Gastroesophageal Reflux Disease Health-Related Quality of Life

Factors			Mean	SD	P value*
BQL score	Pre-operative BMI grade	Class I obesity	42.9	9.0	0.318
		Class II obesity	45.6	4.3	
		Class III obesity	45.7	5.1	
	Current BMI grade	Underweight	44.5	5.3	0.709
		Normal Weight	45.8	5.3	
		Overweight	46.1	4.1	
		Class I obesity	44.4	6.6	
		Class II obesity	43.5	4.2	
		Class III obesity	44.0	4.2	
	Gender	Men	45.1	4.9	0.823
		Women	45.9	5.7	
	EWL%	Less than 50%	42.6	4.7	0.041
		50% - 74.9%	44.4	6.3	
		75% or more	46.0	4.9	
Factors			Median	IQR	P value**
GERD-HRQoL	Pre-operative BMI grade	Class I obesity	10	13	0.689
		Class II obesity	7	12	
		Class III obesity	7	15	
	Current BMI grade	Underweight	17.5	-	0.543
		Normal Weight	7	11	
		Overweight	8	17	
		Class I obesity	9	17	
		Class II obesity	5.5	-	
		Class III obesity	11	-	
	Gender	Men	10	18	0.411
		Women	7	13	
	EWL%	Less than 50%	11	20	0.942
		50% - 74.9%	9	15	
		75% or more	7	14	

Changes in body weight and BMI three years after SG

**Figure 1:**
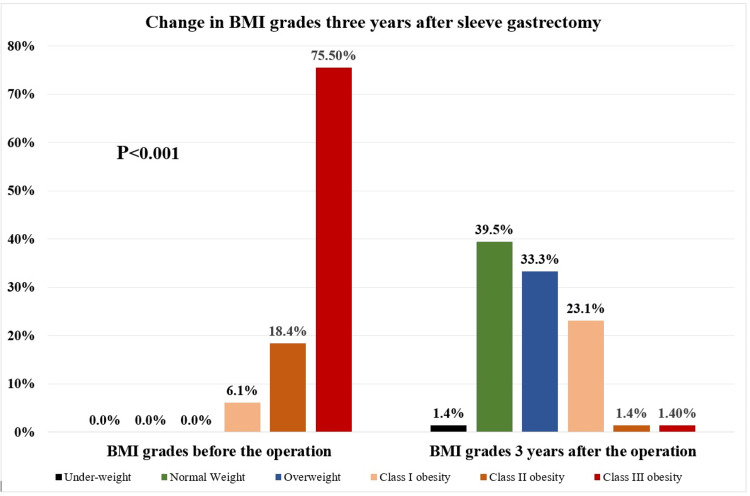
The change in BMI grades three years after sleeve gastrectomy

The mean±SD body weight before operation (124± 23.7 kg) was significantly decreased (P<0.001) to reach 73.6± 14.9 kg three years postoperation with a mean±SD EWL% of 93.4± 26.3% and mean±SD total weight loss TWL% of 39.7±11.2%. Similarly, the mean±SD BMI before operation (45±7.0 kg/m^2^) was significantly decreased to reach 26.8± 4.6 kg/m^2^ three years after operation (P<0.001). The least body weight reached after the operation was collected and the mean±SD value was 68.5±14.2 kg. BMI grades were compared before and three years after gastrectomy where a statistically significant difference (P<0.001) was observed. More details on the change in BMI grades observed three years after the surgery are shown in Table 3 and Figure 1.

**Table 3:** Changes in BMI grade three years after sleeve gastrectomy *Pearson chi-square asymptotic significance (2-sided)

Preoperative BMI grade	Underweight		Normal weight		Overweight		Class I obesity		Class II obesity		Class III obesity		P value
	Count	%	Count	%	Count	%	Count	%	Count	%	Count	%	
Class I obesity (n=9)	1	11.1	6	66.7	2	22.2	0	0.0	0	0.0	0	0.0	<0.001
Class II obesity (n=27)	0	0.0	16	59.3	7	25.9	3	11.1	1	3.7	0	0.0	
Class III obesity (n=111)	1	0.9	36	32.4	40	36.0	31	27.9	1	0.9	1.8	2	

## Discussion

This was a cross-sectional, observational, survey-based study to evaluate the changes in patients’ quality of life and BMI three years post-sleeve gastrectomy. The current study included 147 adult bariatric patients attending the outpatient surgical clinics at KAASH.

It is reported in the literature that patients with morbid obesity usually experience a series of physical changes after getting SG, including significant long-term weight loss, increased satiety, and better energy levels [21]. In the current study, all patients were suffering from class I (6.1%), class II (18.4%), or class III (75.5%) obesity before undergoing SG while three years post-surgery, the vast majority of the patients (72.8%) reached their normal weight (39.5%) or were overweight (33.3%) with a statistically significant difference between the distribution of BMI grades before and after surgery (P<0.001). The reported mean±SD body weight before the operation (124±23.7 kg) reached 73.6±14.9 kg three years post-operation. Similarly, the mean± SD preoperative BMI (45.7±7.0 kg/m2) reached 26.7±4.6 kg/m² post-operation.

In the current study, the mean±SD EWL% was 93.4± 26.3%. This is higher than the values reported in the literature where a systematic review by Fischer et al., including 123 papers with a total population of 12,129 patients reported a mean EWL% of 66.0% after 36 months of SG [22]. Another study was conducted by Hoyuela et al., who reported a mean EWL% of 76.7±21.3% at three years post SG [23]. In addition, the EWL% in our study was higher than the rates reported by the third international summit of sleeve gastrectomy, where the mean EWL% after three years was 64.0% [24]. A study conducted by Berry et al. reported an EWL% of 75.8% and a TWL% of 22.1% post-surgery which is lower than the mean TWL% reported in our study (39.7%) [25]. The study conducted by Mocian and Coroș reported a mean EWL% of 85.1±22.3% [26], which is the nearest to the percent reported in our study.

In our study, the mean± SD BQL score was 45.5±5.2 points (out of 78 points). This is comparable to the results of a multicenter study conducted by Felsenreich et al. to assess the long-term change in the bariatric quality of life 10 years after SG, where the mean total score was 48.2±9.8 points [16].

Our results showed a statistically significant difference between the BQL scores of patients with different EWL% degrees (P=0.041) where patients with EWL% less than 50% had a lower BQL score (42.6± 4.7%) compared to patients with EWL% of 50-74.9% (44.4± 6.3%) and those with EWL% of 75% or more (46.0± 4.9%). This could be related to the results reported by Felsenreich et al. where the BQL score among patients with EWL% <25% (47.8% ± 5.6 points) and those with EWL% of 25.0 to 49.0% (45.9 ± 12.5 points) was lower than the score of those with EWL% of 50% or more (49.8 ± 9.1 points) while the difference they reported was not statistically significant [16].

The median value of the GERD-HRQoL score was 7 out of 50 points with an IQR of 15 points where a lower score indicates better health status (i.e., lower severity of symptoms). The findings are similar to those reported by Althuwaini et al., where patients with high preoperative BMIs were less likely to develop new-onset or worsening symptoms of GERD [27]. The same study by Althuwaini et al. revealed that no variables could predict the development or worsening of GERD symptoms [27], which is consistent with our study where the GERD-HRQoL score was not affected by age, gender, preoperative BMI, current BMI, EWL%, or duration since the operation (P>0.05).

Our study results highlight that the quality of life after SG is better among patients who experienced higher EWL% compared to other patients. In addition, some patients could suffer from GERD with low severity of symptoms. The study also stresses that SG has sustainable mid-term results, as our participants sustained improved BMI for three years postop. The main limitation of our study is that it is a single-institutional study with a small sample size. More multicenter studies on SG with larger participants number are needed to provide more in-depth data on the effects of SG on the patient’s QoL.

## Conclusions

The results of the current study showed that the vast majority of patients undergoing SG lost 75% of their excess body weight and maintained their weight loss for more than three years. In addition, patients with higher percentages of EWL showed a better bariatric quality of life scores after three years of SG compared to other patients with lower percentages of EWL. On the other hand, EWL percentages showed no impact on GERD quality of life.
